# Effects of 4-week astaxanthin supplementation on athletic performance and body composition in young male taekwondo athletes: a randomized, double-blind, placebo-controlled trial

**DOI:** 10.3389/fnut.2025.1731899

**Published:** 2025-12-15

**Authors:** Xiu-Chang Zhang, Meng-Yuan Shu, Ke-Min Li, Ning Wang, Xiao-Yu Wang, Li Shao, Yong Yang, Wei Liu, Shuai Zhu, Li Zuo, Guo-Qi Li, Xiao-Hua Chen, Jian Liang, Chul-Hyun Kim

**Affiliations:** 1 Department of Sports Science, Hengyang Normal University, Hengyang, China; 2 Experimental Teaching Demonstration Center of Food Safety and Nutrition, Xinjiang Institute of Technology, Aksu City, China; 3 Aksu Institute of Apple, Xinjiang Institute of Technology, Aksu City, China; 4 Department of Sports Medicine, Soonchunhyang University, Asan-si, Republic of Korea; 5 Department of Cell Biology, Nanjing Medical University, Nanjing, China; 6 Department of Life Sciences, Hengyang Normal University, Hengyang, China

**Keywords:** astaxanthin, athletes, athletic performance, body composition, Taekwondo

## Abstract

Astaxanthin is an orange-colored natural antioxidant abundantly found in crustaceans and microalgae. It exhibits diverse physiological and protective properties, including anti-inflammatory and antioxidative effects, which make it a promising ergogenic supplement for athletes. This study aimed to investigate the effects of 4 weeks of daily astaxanthin supplementation (12 mg/day) on physical performance and body composition in young male Taekwondo athletes. A randomized, double-blind, placebo-controlled design was employed to ensure the reliability of outcomes. The results showed that astaxanthin supplementation did not produce significant changes in body composition variables such as muscle mass or body fat mass. However, compared with the placebo group, athletes who received astaxanthin exhibited greater improvements in sport-specific kicking performance. Double jump kick performance increased from 67.2 ± 1.8 to 70.1 ± 2.5 kicks/20 s in the astaxanthin group, whereas it remained essentially unchanged in the placebo group (67.9 ± 2.5 to 66.9 ± 2.7 kicks/20 s). Between-group differences in double jump kick performance became statistically significant from week 1 onward and were maintained through week 4 (*p* < 0.05). Similarly, high roundhouse kick performance improved from 93.2 ± 5.0 to 96.7 ± 4.9 kicks/60 s in the astaxanthin group, compared with 94.7 ± 3.2 to 93.6 ± 3.1 kicks/60 s in the placebo group, with significant between-group differences first detected at week 2 and persisting until week 4 (*p* < 0.05). These findings suggest enhanced anaerobic capacity and neuromuscular coordination. No adverse effects or gastrointestinal discomfort were reported during the supplementation period, indicating that astaxanthin was well-tolerated. In conclusion, short-term daily supplementation with 12 mg of astaxanthin can safely and effectively improve specific performance parameters in Taekwondo athletes. We recommend continuous intake for at least 2 weeks before competition to optimize performance outcomes.

## 1 Introduction

Astaxanthin is a naturally occurring lipophilic orange carotenoid predominantly found in microalgae, fish, and crustaceans and has been commercially utilized mainly in the feed industry and dietary supplements since it was first isolated from lobster in 1938 (1, 2). Astaxanthin exhibits multiple physiological and therapeutic properties, including anti-inflammatory and antioxidative effects, attenuation of skin aging, and potential benefits for cardiometabolic and neurological disorders (3–6). It also plays a regulatory role in hyperlipidemia and diabetes, enhances immune function, and improves muscular endurance (7, 8). As a potent antioxidant, astaxanthin can act directly on mitochondria to modulate energy metabolism, thereby contributing to improved athletic performance (9, 10). The restoration of damaged muscle tissue is facilitated through the delivery of functionally active mitochondria, which promotes muscle repair and reduces fibrosis (11–13). In addition, enhanced blood flow supports the clearance of metabolic by-products and increases oxygen and nutrient supply to skeletal muscles, resulting in better exercise performance (14). Supplementation with astaxanthin has been shown to improve overall mitochondrial health, mitigate oxidative stress, and upregulate the expression of anti-inflammatory transcription factors and antioxidant enzymes (15–17). Considering its broad range of physiological benefits, astaxanthin supplementation has been extensively examined for its potential to enhance athletes' performance, accelerate recovery, and modulate physiological adaptations throughout training and competition.

Taekwondo, originating from the Korean Peninsula and officially introduced as an Olympic sport in 2000 (18, 19), is characterized by repeated bouts of short-term, near-maximal intensity efforts interspersed with brief recovery periods (20, 21). The sport emphasizes kicking techniques, which account for more than 70% of scoring actions, requiring athletes to perform rapid and explosive lower-limb movements such as kicking, dodging, and counterattacking (22, 23). These repeated high-intensity offensive and defensive exchanges place heavy demands on lower-body strength, speed, agility, and cardiorespiratory endurance.

Given the intermittent and high-intensity nature of Taekwondo, several studies have evaluated the ergogenic effects of various nutritional supplements. For instance, caffeine supplementation has been shown to improve reaction time, kicking frequency, and perceived exertion thresholds in Taekwondo athletes (24). Similarly, beetroot juice, rich in dietary nitrates, can enhance intermittent performance by promoting nitric oxide synthesis, improving vasodilation, and increasing muscle oxygenation (25, 26). However, the magnitude of these effects appears to vary depending on individual characteristics, supplementation dosage, and training status (27).

Astaxanthin has been investigated in various sports disciplines because of its potential to enhance endurance, attenuate oxidative stress and facilitate post-exercise recovery. For example, a 28 day intervention with 4 mg per day improved cycling time trial performance in trained young adults (28). In another study, a 4 week protocol providing 12 mg per day to resistance trained men significantly reduced subjective markers of delayed onset muscle soreness, although it did not produce measurable gains in strength output (29). In addition, an 8 mg per day regimen over 4 weeks was reported to support the recovery of immune related plasma proteins following prolonged running (30). Collectively, these findings suggest that astaxanthin supplementation can provide performance related and physiological benefits in different athletic populations. However, no study has examined its effects in Taekwondo athletes, particularly in relation to sport specific performance outcomes or changes in body composition. Previous research indicates that short term supplementation from 1 to 4 weeks may already be sufficient to induce beneficial adaptations in athletes (1, 28, 31). Based on this evidence, the present study aimed to evaluate the ergogenic and physiological effects of a 4 week astaxanthin supplementation in young male Taekwondo athletes.

## 2 Materials and methods

### 2.1 Recruitment and characteristics of participants

The present experiment commenced in June 2025 and recruited a total of 42 male Taekwondo athletes from a professional sports team in Hengyang, Hunan Province, China. Participants were selected according to the following inclusion criteria: (1) aged between 15 and 18 years; (2) engaged in Taekwondo training for at least 1 year; (3) had prior experience in municipal or higher-level competitions; and (4) non-smokers and non-drinkers. All participants were in good general health and free from any acute or chronic diseases that could interfere with exercise performance or supplementation effects.

### 2.2 Ethical approval and informed consent

This study was reviewed and approved by the Institutional Ethics Committee of Hengyang Normal University (Approval No. 20251110) in accordance with the Declaration of Helsinki. All participants and their legal guardians were fully informed about the objectives, procedures, and potential risks of the study prior to enrollment. Written informed consent was obtained from both the athletes and their guardians before participation. Personal information of all participants was kept confidential and used solely for research purposes.

### 2.3 Experimental design

This study employed a randomized, double-blind, placebo-controlled design to examine the effects of astaxanthin supplementation on body composition and exercise performance in male Taekwondo athletes. A total of 42 participants were randomly assigned to either the experimental group (receiving astaxanthin capsules) or the control group (receiving placebo capsules) in a 1:1 ratio using a computer-generated randomization schedule created in Microsoft Excel 2021 (Microsoft Corp., Redmond, WA, USA). Both participants and investigators were blinded to the group allocation throughout the study period.

The intervention lasted for 4 weeks. Participants in the experimental group consumed 12 mg of astaxanthin once daily in the morning, while those in the control group received identical placebo capsules containing sunflower seed oil. The astaxanthin capsules were supplied by Nutricost (Nutricost Inc., Vineyard, UT, USA) and contained only 12 mg of natural astaxanthin and sunflower seed oil as the carrier. All athletes maintained their regular Taekwondo training routines throughout the study, consisting of five sessions per week, with each session lasting ~120 min, including technical skill drills, sparring practice, and physical conditioning.

Compliance was monitored through weekly capsule counts and training attendance logs. Participants were instructed to maintain their habitual diet and refrain from taking any additional nutritional supplements or medications during the intervention period. The use of any extra antioxidant or vitamin supplements other than those provided in the current study was strictly prohibited. Additionally, participants were provided with a list of astaxanthin-rich foods to avoid in order to minimize potential dietary confounding. To ensure accurate compliance, supplementation was strictly supervised by the team coach. Capsules were consumed each morning under direct supervision, and attendance at all training sessions was continuously monitored by coaching staff. As a result, capsule intake and training attendance both reached 100% across all participants throughout the 4-week intervention, with no deviations reported.

**Figure 1:**
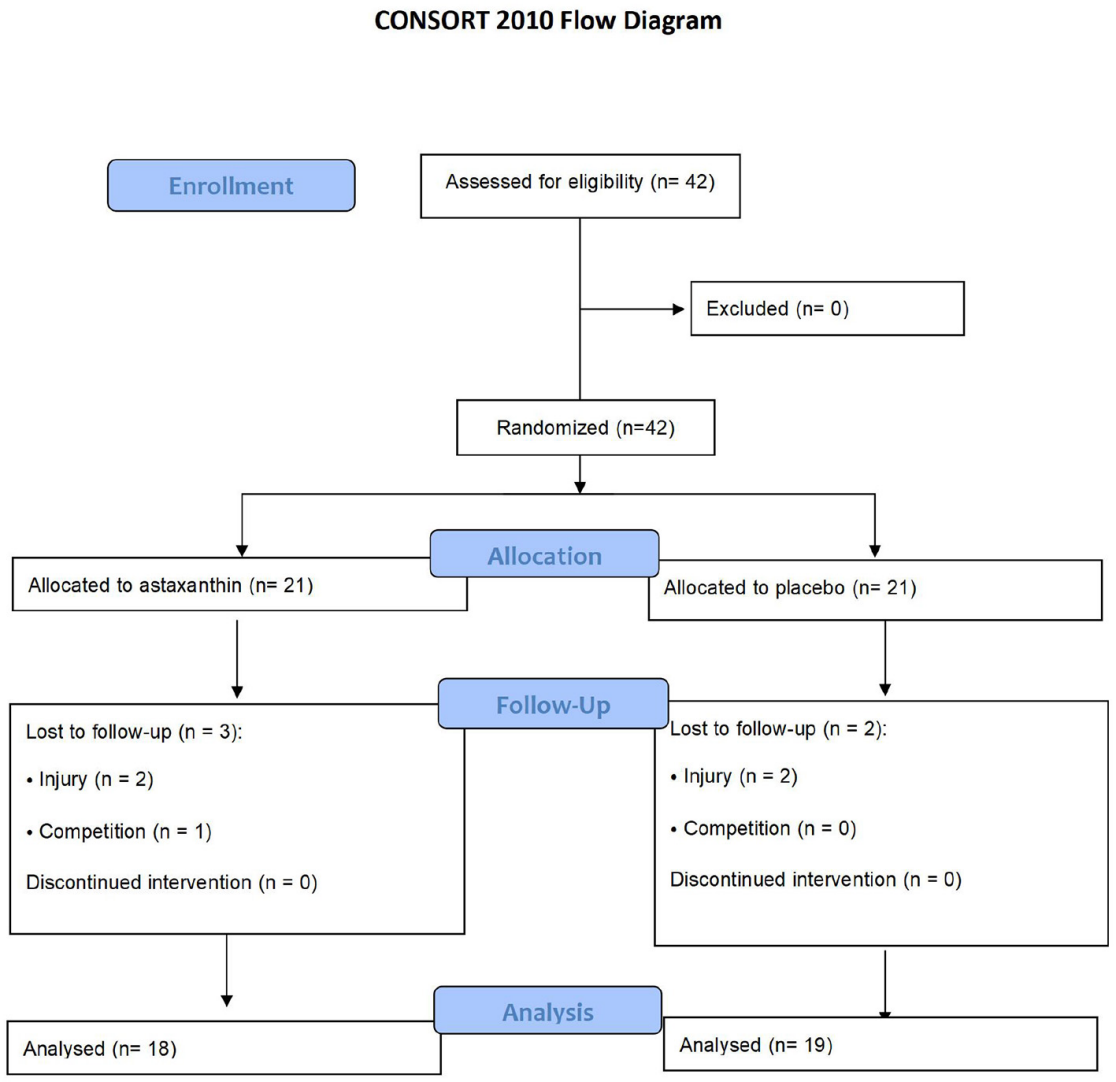
CONSORT 2010 flow diagram showing participant enrollment, randomization, allocation, follow-up, and analysis for the astaxanthin and placebo groups.

A total of 42 male Taekwondo athletes were initially randomized, with 21 assigned to the experimental group and 21 to the control group. During the study, three participants in the experimental group were excluded (two due to injury and one due to competition). In comparison, two participants in the control group withdrew because of injury. Consequently, 18 participants in the experimental group and 19 participants in the control group were included in the final analysis. The baseline characteristics, including age, height, and body weight, of the participants in both groups are presented in Table 1, and the participant flow throughout the study is illustrated in Figure 1.

**Table 1:** Baseline characteristics (mean ± SD) of male Taekwondo athletes in the experimental and control groups.

**Group**	**Age (year)**	**Weight (kg)**	**High (cm)**
Experimental group	15–17 (16.38 ± 0.98)	52–95 (78.25 ± 4.71)	168–184 (176.8 ± 4.71)
Control group	15–18 (16.05 ± 0.83)	51.7–105 (76.41 ± 15.99)	165–190 (176.7 ± 7.42)

### 2.4 Body composition and exercise performance tests

Body composition was evaluated using a bioelectrical impedance analyzer (Jinghai S-one+5s, Jinhai Inc., Beijing, China) to determine muscle mass (kg), intracellular fluid (kg), extracellular fluid (kg), and body fat mass (kg). Measurements were conducted on weekend mornings each week under standardized conditions, with participants fasting for at least 8 h and abstaining from strenuous exercise for 24 h before testing. Baseline assessments were taken the day before Week 1. All measurements were performed by the same trained technician to ensure consistency and reliability.

Exercise performance tests were conducted immediately after body composition assessments. Participants first performed a 20-s double jump kick test using a Leda A1005 device (Leda Inc., Nanjing, China) to evaluate explosive power. Following a 10-min recovery period, they completed a 60-s high roundhouse kick test using the Leda A1006 device to assess muscular endurance. These test durations were selected based on common performance evaluation protocols in Taekwondo. Short-duration kick tests of ~20 s are widely used to assess anaerobic power and movement speed, and are comparable to the Frequency Speed of Kick Test (FSKT), which has demonstrated good test–retest reliability (ICC = 0.74–0.89) in trained Taekwondo athletes (32). Similarly, high-intensity kicking tests lasting 40–60 s have been reported to reflect anaerobic capacity and fatigue resistance in Taekwondo athletes, supporting the validity of using a 60-s kicking protocol to assess muscular endurance (33). Throughout all tests, standardized verbal encouragement was provided to ensure maximal effort. To minimize fatigue-related bias, a minimum of 24 h of recovery was maintained between high-intensity training sessions and testing days.

### 2.5 Adverse event monitoring

Throughout the study, potential adverse events were closely monitored to ensure participant safety and intervention tolerability. Any physical discomfort, gastrointestinal symptoms, allergic reactions, or other unexpected health issues reported by participants were documented and evaluated. Adverse events were recorded using the NIH Adverse Event Reporting Template (https://files.nccih.nih.gov/s3fs-public/CR-Toolbox/Adverse_Event_Form_ver2_07-17-2015.pdf).

Participants were instructed to report any unusual symptoms to the research team promptly. Additionally, trained medical personnel conducted weekly interviews during the assessment sessions to identify any unreported events. No adverse events or side effects were observed throughout the experimental period.

### 2.6 Statistical analysis

All statistical analyses were conducted by an independent analyst who was blinded to group allocation and not involved in the experimental procedures. To examine differences in body composition variables and exercise performance between groups, the Shapiro–Wilk test was first used to assess the normality of data distribution. When the data satisfied the assumption of normality, an independent samples *t*-test was employed to compare differences between the experimental and control groups. If the assumption of normality was not met, a nonparametric Mann–Whitney *U* test was applied.

In addition to group comparisons, the longitudinal structure of the dataset was considered. Because each participant was assessed repeatedly at five time points (baseline and Weeks 1–4), the data followed a repeated-measures design. To account for this temporal dependency and potential group × time interactions, a permutational multivariate analysis of variance (PERMANOVA) was conducted using Week and Group as fixed factors, with 999 permutations of the residuals under a reduced model. Prior to PERMANOVA, body composition and performance data were log-transformed [log(x + 1)] and normalized to reduce heteroscedasticity and scale differences across variables. Statistical significance was set at *p* < 0.05. All statistical analyses and graphical visualizations were performed using R software version 4.0 (R Foundation for Statistical Computing, Vienna, Austria).

## 3 Results

### 3.1 Changes in body composition

Table 2 summarizes the changes in body composition parameters of the experimental and control groups at baseline and during the 4-week intervention period. At baseline, there were no significant differences between the experimental and control groups in muscle mass, intracellular fluid, extracellular fluid, or body fat mass, as determined by the Mann–Whitney *U* test (*p* > 0.05).

**Table 2:** Changes in body composition of the experimental and control groups at baseline and during the intervention weeks.

**Body composition**	**Group**	**Baseline**	**Week 1**	**Week 2**	**Week 3**	**Week 4**
Muscle mass (Kg)	Experimental group	44.5–67.1 (52.7 ± 6.1)	44.2–66.3 (52.9 ± 5.8)	43.0–65.2 (52.5 ± 5.6)	43.3–65.9 (52.8 ± 5.9)	42.6–65.9 (52.5 ± 5.7)
	Control group	46.4–58.8 (52.5 ± 3.8)	46.9–59.6 (52.5 ± 4.1)	46.3–59.4 (52.4 ± 3.7)	46.7–59.7 (52.5 ± 4.0)	45.7–60.6 (52.8 ± 4.4)
Intracellular fluid (Kg)	Experimental group	23.5–34.0 (27.5 ± 2.8)	23.5–33.7 (27.6 ± 2.7)	23.1–33.2 (27.5 ± 2.6)	23.3–33.7 (27.5 ± 2.8)	23.0–33.6 (27.5 ± 2.7)
	Control group	24.4–30.3 (27.4 ± 1.9)	24.5–30.5 (27.3 ± 2.0)	24.1–30.7 (27.4 ± 1.8)	24.0–30.6 (27.3 ± 2.0)	24.0–30.8 (27.4 ± 2.1)
Extracellular fluid (Kg)	Experimental group	10.5–18.8 (13.57 ± 2.24)	10.4–18.3 (13.6 ± 2.1)	10.0–17.9 (13.4 ± 2.0)	10.0–18.0 (13.7 ± 2.0)	9.7–18.1 (13.4 ± 2.0)
	Control group	11.8–15.9 (13.6 ± 1.3)	11.8–16.5 (13.7 ± 1.4)	11.7–15.9 (13.5 ± 1.2)	12.0–16.6 (13.8 ± 1.3)	11.6–17.2 (13.8 ± 1.6)
Body fat mass (Kg)	Experimental group	1–25.5 (7.7 ± 6.0)	1.0–25.6 (7.4 ± 6.3)	1.0–25.3 (7.2 ± 6.2)	1.0–26.4 (7.5 ± 6.5)	1.0–27.6 (7.3 ± 6.6)
	Control group	1.1–21.9 (8.9 ± 5.9)	1.2–22.1 (8.4 ± 5.9)	1.3–21.0 (8.8 ± 5.6)	1.1–24.4 (8.5 ± 6.2)	1.1–22.2 (8.9 ± 5.8)

Values are expressed as mean ± standard deviation (Mean ± SD); Muscle mass, intracellular fluid, extracellular fluid, and body fat mass were measured at baseline and at the end of each intervention week (Weeks 1–4).

Throughout the entire intervention period, all measured body composition variables remained relatively stable in both groups. Although slight fluctuations in muscle mass and body fat mass were observed in the experimental group, these changes were not statistically significant compared with the control group (Mann–Whitney *U* test, *p* > 0.05). Similarly, intracellular and extracellular fluid levels remained consistent across all testing weeks (Mann–Whitney *U* test, *p* > 0.05), indicating that astaxanthin supplementation did not significantly affect the athletes' hydration status or tissue composition during the 4-week intervention period.

### 3.2 Changes in exercise performance

**Figure 2:**
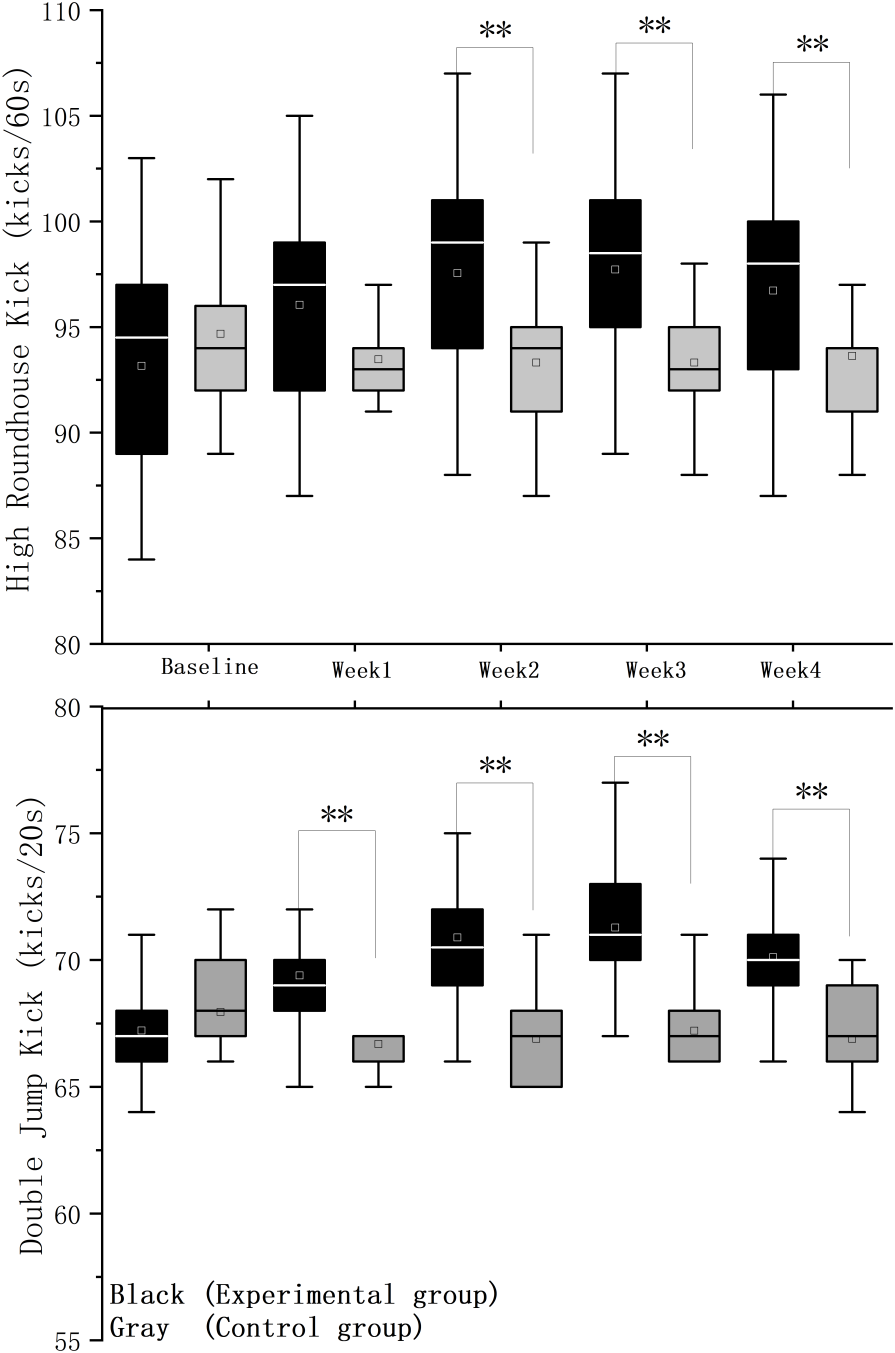
Comparison of kicking performance between the experimental group (black) and the control group (gray) across 4 weeks. **p < 0.01.

Figure 2 illustrates the changes in high roundhouse kick (60 s) and double jump kick (20 s) performance in the experimental and control groups over the 4-week intervention period. At baseline, there were no significant differences between the two groups in either performance variable (Mann–Whitney *U* test, *p* > 0.05). During the intervention, the experimental group showed a slight upward trend in both performance indicators, whereas the control group remained relatively stable. However, as the intervention progressed, significant between-group differences emerged. The experimental group demonstrated a significant improvement in double jump kick performance compared with the control group during Weeks 1 (Mann–Whitney *U* test, *z* = 3.04, *r* = 0.50, *p* = 0.002), 2 (Mann–Whitney *U* test, *z* = 4.58, *r* = 0.70, *p* = 0), 3 (Mann–Whitney *U* test, *z* = 4.38, *r* = 0.72, *p* = 0), and 4 (Mann–Whitney *U* test, *z* = 3.43, *r* = 0.56, *p* = 0.001). Similarly, performance in the high roundhouse kick test showed a significant increase in the experimental group relative to the control group during Weeks 2 (Mann–Whitney *U* test, *z* = 2.79, *r* = 0.45, *p* = 0.005), 3 (Mann–Whitney *U* test, *z* = 2.94, *r* = 0.48, *p* =0.003), and 4 (Mann–Whitney *U* test, *z* = 2.17, *r* = 0.36, *p* = 0.03). A PERMANOVA with Group and Week as fixed factors revealed a significant Group × Week interaction (Pseudo-*F* = 4.4, *p* = 0.001), indicating that the effects of astaxanthin supplementation varied over time (Table 3).

**Table 3:** PERMANOVA results for the effects of astaxanthin supplementation and time on Taekwondo kicking performance (double jump kick and high roundhouse kick).

**Source**	**Degrees of freedom**	**Sum of squares**	**Mean square**	**Pseudo-*F***	***p* (perm)**
Week	4	10.7	2.7	1.7	0.111
Group	1	53.341	53.341	33.705	0.001
Group × week	3	276.9	6.9	4.4	0.001

## 4 Discussion

In our 4-week intervention study involving young male Taekwondo athletes who received a daily supplementation of 12 mg of astaxanthin, athletic performance showed a significant improvement (*p* < 0.01), whereas body composition did not exhibit any notable changes (*p* > 0.05). The ergogenic effects of astaxanthin on athletes have been well-documented in numerous experimental studies. For instance, daily supplementation with 12 mg of astaxanthin for 7 days led to a 1.2% reduction in completion time during a 40 km cycling time trial (*p* = 0.029), accompanied by a significant increase in whole-body fat oxidation during the final stage of exercise (*p* = 0.044) (31). The present results are consistent with previous reports demonstrating improved endurance following short-term astaxanthin supplementation.

However, unlike cycling or running, Taekwondo involves repeated bouts of high-intensity anaerobic movements interspersed with brief recovery periods, which may influence the onset and magnitude of astaxanthin's ergogenic effects. In this study, the onset of ergogenic effects varied according to the type of performance test. An improvement in the high roundhouse kick (60 s) was observed after 2 weeks of astaxanthin supplementation, whereas an enhancement in the double jump kick (20 s) appeared earlier. The early improvement in the double jump kick may be attributed to rapid enhancements in mitochondrial redox balance and ATP turnover induced by astaxanthin supplementation (34–36). Astaxanthin enhances mitochondrial membrane integrity and reduces ROS generation, thereby maintaining ATP production efficiency during repeated high-intensity efforts (37, 38). In contrast, the high roundhouse kick requires sustained muscular contractions and coordination, and its performance gain may depend on longer-term neuromuscular adaptations and greater efficiency in energy substrate utilization, which typically emerge after a more extended supplementation period. Moreover, the energy system demands of these two tasks differ substantially. The double jump kick primarily depends on the phosphagen and anaerobic glycolytic pathways, whereas the high roundhouse kick performed continuously for 60 s relies more heavily on aerobic metabolism. Therefore, the antioxidant and mitochondrial regulatory functions of astaxanthin may influence these energy systems on different temporal scales (39). The delayed improvement observed in the high roundhouse kick may also reflect the time required for astaxanthin to accumulate within skeletal muscle, where it helps mitigate exercise-induced oxidative stress and supports post-exercise recovery (40, 41). Such time-dependent effects may correspond to the gradual incorporation of astaxanthin into muscle cell membranes and mitochondrial structures, enabling sustained redox homeostasis during prolonged activity (4, 6).

Although the present study demonstrated that astaxanthin supplementation did not significantly affect body composition in Taekwondo athletes, current evidence regarding its impact on body composition indicators remains inconsistent. Some interventions that combined supplementation with exercise training have reported favorable changes. For instance, 12 weeks of high-intensity training combined with daily supplementation of 20 mg astaxanthin significantly reduced body weight and body fat in obese subjects while improving several cardiovascular risk factors (42). Similarly, a 4-month functional training study in older adults aged 65–82 years found that participants receiving a formulation containing astaxanthin (12 mg), tocotrienol, and zinc showed marked increases in maximal voluntary contraction, muscle cross-sectional area, and muscle strength per unit area, whereas no significant changes were observed in the control group (43). However, studies using astaxanthin supplementation alone have generally reported no significant effects. Heidari et al. conducted an 8-week randomized controlled trial involving 50 patients with coronary artery disease. They found no statistically significant differences between the astaxanthin and placebo groups in changes in body weight, BMI, or body fat percentage (44). A recent meta-analysis that pooled data from nine clinical trials also reported no overall effect of astaxanthin on BMI or body weight (45). These discrepancies may be related to differences in supplementation duration, dosage, population characteristics and the presence or absence of concurrent exercise training. Since adaptations in body composition typically require a longer period along with resistance-based training, the 4-week intervention in the present study may have been too short to induce measurable changes. Therefore, the mechanisms by which astaxanthin may influence body composition should be regarded as potential hypotheses that require further exploration, rather than confirmed physiological effects. Future studies should consider extending the intervention period to 8–12 weeks or longer, incorporating structured resistance training, and examining biochemical indicators to clarify whether astaxanthin can modulate body composition in athletic populations.

In terms of safety and tolerability, a daily supplementation of 12 mg of astaxanthin for 4 weeks was well-tolerated by young male athletes, with no adverse effects reported, including mild symptoms such as gastrointestinal discomfort or other minor complaints. A human clinical review covering more than 100 studies also concluded that no clinical safety issues were reported, whether in short-term studies involving daily intake of up to 24 mg for 12 weeks or in long-term studies lasting more than 6 months (46). However, only a small portion of studies investigating performance-enhancing supplements in athletes have systematically monitored adverse effects (47). This lack of safety assessment limits the ability to fully evaluate the balance between benefits and potential risks. Even minor or temporary side effects such as gastrointestinal discomfort, sleep disturbance, or fatigue may negatively influence training quality and competitive performance. Therefore, it is essential to include comprehensive adverse event monitoring within the experimental design and evaluation framework of future studies on dietary supplements for athletes.

Although strong dietary deviations were unlikely due to the uniform training schedule and cafeteria-based meal routines, the absence of systematic dietary monitoring remains a limitation and may have masked subtle performance or recovery-related effects. Compliance was monitored through capsule counts and training attendance logs, but future studies should incorporate structured dietary tracking, for example 24-h food records, dietary recall questionnaires, or nutrient intake biomarkers to objectively verify compliance and further minimize potential confounding.

Several other limitations should be noted. First, the study involved only adolescent male Taekwondo athletes, which may limit the generalizability of the findings to other populations or athletic disciplines. Second, although the 4-week intervention period may appear short, it was based on previous research showing that short-term astaxanthin supplementation (1–4 weeks) may produce beneficial effects on athletic performance (1, 28, 31). The present findings partly support this notion, yet longer intervention periods are needed to verify the durability and magnitude of these effects. Nevertheless, this time frame may have been insufficient to capture long-term adaptations in body composition or aerobic capacity, which typically require extended training periods. Third, the mechanistic explanations regarding redox regulation, neuromuscular efficiency, and energy substrate utilization remain hypothetical because no direct biochemical markers were measured in this study. Future research should incorporate molecular-level assessments, larger sample sizes, and longer supplementation durations, for instance more than 8–12 weeks to determine both acute and chronic responses to astaxanthin. The application of advanced analytical techniques such as metabolomic profiling, muscle oxygenation monitoring, and near-infrared spectroscopy may provide deeper insights into how astaxanthin modulates energy metabolism, fatigue resistance, and exercise recovery in combat sport athletes. Finally, combining astaxanthin supplementation with structured resistance training and examining dose–response relationships will help clarify whether astaxanthin can enhance both aerobic and anaerobic performance through mechanisms such as mitochondrial biogenesis, lipid metabolism modulation, or reduced inflammatory signaling.

## 5 Conclusions

This randomized, double blind, placebo-controlled trial demonstrated that daily supplementation with 12 mg of astaxanthin for four consecutive weeks can significantly improve specific performance parameters in young male Taekwondo athletes, particularly those related to anaerobic capacity and neuromuscular coordination, such as the double jump kick and high roundhouse kick. However, no significant changes in body composition were observed throughout the intervention period. The findings suggest that astaxanthin may exert time dependent ergogenic effects, with earlier benefits observed in explosive performance tasks and delayed improvements in endurance related activities. Additionally, the supplement was well-tolerated, and no adverse events were reported, supporting its short-term safety for use in adolescent athletes. While these results are encouraging, further research is needed to confirm the ergogenic potential of astaxanthin across diverse athletic populations and to explore its long-term effects on metabolic, physiological, and molecular adaptations. Future studies should adopt larger sample sizes, extend the duration of supplementation, and incorporate biochemical and imaging-based assessments to elucidate the underlying mechanisms. Moreover, evaluating dose response relationships and including female athletes as well as other combat sport disciplines may enhance the generalizability of the findings and support evidence-based recommendations for the use of astaxanthin in athletic settings.

## Data Availability

The raw data supporting the conclusions of this article will be made available by the authors, without undue reservation.
